# Wasabi Compound 6-(Methylsulfinyl) Hexyl Isothiocyanate Induces Cell Death with Coexisting Mitotic Arrest and Autophagy in Human Chronic Myelogenous Leukemia K562 Cells

**DOI:** 10.3390/biom9120774

**Published:** 2019-11-23

**Authors:** Kun-Ming Wu, Hui-Fen Liao, Chih-Wen Chi, Yu Ru Kou, Yu-Jen Chen

**Affiliations:** 1 Institute of Physiology, School of Medicine, National Yang-Ming University, Taipei 11221, Taiwan; chest4080@yahoo.com.tw; 2 Chest Division, Department of Internal Medicine, MacKay Memorial Hospital, Taipei 10449, Taiwan; 3 Mackay Junior College of Medicine, Nursing, and Management, Taipei 25245, Taiwan; 4 Department of Biochemical Science and Technology, National Chiayi University, Chiayi 60004, Taiwan; liaohf@mail.ncyu.edu.tw; 5 Department of Medical Research, MacKay Memorial Hospital, New Taipei City 25160, Taiwan; cwchid48906003@gmail.com; 6 Department of Nursing, MacKay Medical College, New Taipei City 25245, Taiwan; 7 Department of Medical Research, China Medical University Hospital, Taichung 40402, Taiwan; 8 Department of Radiation Oncology, MacKay Memorial Hospital, Taipei 10449, Taiwan

**Keywords:** wasabi, 6-(methylsulfinyl) hexyl isothiocyanate, mitosis, autophagy, chronic myelogenous leukemia

## Abstract

A natural compound from *Wasabia japonica*, 6-(methylsulfinyl) hexyl isothiocyanate (6-MITC) was investigated for its anti-leukemia activity and mechanism of action. It was found that 6-MITC inhibited the viability of human chronic myelogenous leukemia K562 cells along with extensive mitotic arrest, spindle multipolarity, and cytoplasmic vacuole accumulation. The evidence of autophagy included the validation of autophagosomes with double-layered membranes under transmission electron microscopy, LC3I/II conversion, and the induction of G2/M phase arrest observed with acridine orange staining of treated cells, as well as the elevation of phosphorylated-histone H3 expression at the M phase. With regard to the expression of proteins related to mitosis, the down regulation of p-CHK1, p-CHK2, p-cdc25c, and p-cdc2, as well as the upregulation of cyclin B1, p-cdc20, cdc23, BubR1, Mad2, and p-plk-1 was observed. The knockdown of cdc20 was unable to block the effect of 6-MITC. The differentiation of k562 cells into monocytes, granulocytes, and megakaryocytes was not affected by 6-MITC. The 6-MITC-induced unique mode of cell death through the concurrent induction of mitosis and autophagy may have therapeutic potential. Further studies are required to elucidate the pathways associated with the counteracting occurrence of mitosis and autophagy.

## 1. Introduction

Chronic myeloid leukemia (CML) is a hematological neoplasm characterized by a gene translocation between chromosome 9q34 and 22q11.2 [1,2]. The fusion of the Abelson gene (Abl) on chromosome 9 and breakpoint cluster region (Bcr) on chromosome 22 results in Bcr-Abl oncoprotein expression. This gene rearrangement is called the Philadelphia chromosome. The Bcr-Abl expression in leukemia, especially in CML and Philadelphia chromosome-positive acute lymphoblastic leukemia (ALL) activates tyrosine kinase and promotes abnormal blood cell growth [3]. The current treatment modalities for the different phases of CML include chemotherapy, interferon, stem cell transplantation, differentiation induction, and target therapy [4,5]. Among them, targeted therapy is the most promising, with minimal complications. Targeted therapy aimed at the tyrosine kinase to block Bcr-Abl transcript expression is currently a pivotal strategy for CML treatment among patients who are ineligible for bone marrow transplantation. The first-line drug developed for CML targeted therapy against tyrosine kinase is imatinib mesylate (IM, also named STI 571) [6]. Imatinib mesylate is an Abl-specific tyrosine kinase inhibitor that selectively abrogates the ATP binding site of Bcr-Abl [3,7,8]. However, several clinical issues are associated with IM therapy in CML, including the resistance or non-tolerance of CML patients with Bcr-Abl mutations to IM treatment [9,10,11]. Resistance to IM in CML is a critical issue in clinical practice, with estimated four-year resistance rates of 20% in the later chronic phase and 70–90% in the accelerated-blastic phases [12,13,14]. Therefore, there is an urgent need to develop novel drugs other than Bcr-Abl inhibitors for CML treatment. 

Autophagy is characterized by the degradation of intracellular components within lysosomes or vacuoles [15]. The process occurs at both the basal level and in response to stress. Basal-level autophagy plays a role in protein and organelle turnover, and is rapidly upregulated when cells need to generate intracellular nutrients and energy. Autophagy and apoptosis are important for the control of the turnover of organelles and proteins in cells. Autophagy generally blocks the induction of apoptosis, and apoptosis-associated caspase activation turns off the autophagic process. However, autophagy may also induce apoptosis or necrosis and lead to autophagic cell death [16].

A naturally-occurring compound, 6-(methylsulfinyl) hexyl isothiocyanate (6-MITC), was isolated from *Wasabia japonica* (wasabi), a pungent spice widely used in Japanese food. Several biological activities of 6-MITC have been reported, such as anti-inflammatory [17,18], neuroprotective [19,20], anti-cancer [21,22,23], and chemopreventive effects [24,25]. Our previous publication indicated that 6-MITC and its derivatives may have an inhibitory effect against pancreatic cancer cell growth (including cancer stem cells phenotype) [26].

In this study, we examined the effect of 6-MITC, a compound isolated from wasabi, on the cell death of human CML cells. In particular, we investigated the mode of cell death and the signaling pathways associated with the compound.

## 2. Materials and Methods

### 2.1. Reagents

Pure 6-MITC was purchased from LKT Laboratories (St. Paul, MN, USA), dissolved in dimethyl sulfoxide (DMSO) (Merck, Darmstadt, Germany), and stored at −20 °C.

### 2.2. Cell Culture

Human chronic myeloid leukemia K562 cells (IM-sensitive and Bcr-Abl^+^) and K562R (IM-resistant and Bcr-Abl^+^) were obtained from the American Type Culture Collection (ATCC) and maintained in a RPMI 1640 medium (Gibco, Grand Island, NY, USA) supplemented with 10% fetal bovine serum (Gibco) and 2 mM L-glutamine (Sigma-Aldrich, St. Louis, MO, USA). Cells were subcultured every two to three days and maintained in an exponential growth state.

### 2.3. Cell Viability

K562 cells were treated with various concentrations of 6-MITC, or pre-treated with 3-methyladenine (3-MA) for 1 h, followed by treatment with 6-MITC for 24 h and 48 h. After treatment, the cells were harvested and the numbers of viable cells were estimated by trypan blue dye exclusion assay.

### 2.4. Cell Cycle Analysis by Flow Cytometry

DNA staining was carried out using BD Cycletest^TM^ Plus DNA reagent kit (BD Biosciences, Franklin Lakes, NJ, USA) according to the manufacturer’s protocol. Briefly, after 6-MITC or 3-MA plus 6-MITC treatment, cells were harvested and fixed with 70% ethanol at 4 °C for 1 h. Cells were incubated with solutions B and C containing 0.1 mg/mL RNase and 0.5 mg/mL propidium iodide (PI) for 10 min. Samples were then filtered using 50-µm nylon mesh and the FACScaliber flow cytometer (Becton Dickinson, Lincoln Park, NJ, USA) was used to analyze the DNA histogram. Data from 10^4^ cells were acquired and analyzed using the ModFit software (Becton Dickinson).

### 2.5. Detection of Phosphorylated Histone H3

The treated cells were collected, fixed in 2% paraformaldehyde, permeabilized with 1% Triton X-100 (Sigma-Aldrich), and stained with anti-phospho-histone H3 (Ser 10)-fluorescein isothiocyanate (FITC) (Cell Signaling, Danvers, MA, USA) at 25 °C for 1 h. The cells were washed with phosphate-buffered saline (PBS) and resuspended in solutions B and C containing PI and RNase A. The samples were subjected to flow cytometry, and the data were analyzed using the CellQuest ^Pro^ software (Becton Dickinson).

### 2.6. Morphology by Light and Electronic Microscopy

For light microscopic examination, the cells were stained by Liu’s stain method using Liu A solution for 45 s followed by the addition of Liu B solution for 90 s on slides. The slides were gently washed and dried, and the cell morphology was observed under a light microscope (Olympus, Tokyo, Japan) at a magnification of 1000×. For transmission electron microscopy (TEM), cells were collected, washed, and fixed with 2.5% glutaraldehyde in cacodylate buffer for 30 min. Samples were then fixed in osmium tetroxide (1%) and embedded in Epon resin (Electron microscopy science, Hatfieldcity, PA, USA). Semithin sections prepared for ultrathin sections were cut, stained with 0.5% toluidine blue, and examined under a light microscope. Ultrathin sections were then stained with 2% uranyl acetate and Reynold’s lead citrate, and observed under a TEM equipped with digital camera (JEM-1200EXII, JEOL Co., Tokyo, Japan).

### 2.7. Immunofluorescent Stain

Cells were harvested, fixed in 4% paraformaldehyde for 10 min, and permeabilized in 1% Triton-X-100 in PBS. After serial washes in PBS, cells were incubated in 10% bovine serum albumin and incubated with anti-α-tubulin (Zymed Laboratories, San Francisco, CA, USA) or anti-γ-tubulin monoclonal antibody (mAb) (Covance, Princeton, NJ, USA) with 1:100 dilutions. Cells were washed in PBS, followed by incubation with cyanine Cy^TM^2-conjugated anti-mouse IgG from donkey or RhodamineRed^TM^-X-conjugated goat anti-rabbit IgG and diluted at 1:100 as secondary antibody (Jackson ImmunoResearch Laboratories, Inc. West Grove, PA, USA). Cells were then incubated in Hoechst 33342 (Sigma-Aldrich) to identify cell nuclei.

### 2.8. Detection of Acidic Vesicular Organelles with Acridine Orange Staining

To quantify the development of acidic vesicular organelles, we stained cells with acridine orange (10 ng/mL) for 15 min, after which the cells were subjected to flow cytometry. In cells stained with acridine orange, the cytoplasm and nucleoli emit green fluorescence, whereas the acidic compartments emit red fluorescence; the intensity is proportional to the degree of acidity. Green (510–530 nm) and red (650 nm) fluorescence emission from 10^4^ cells illuminated with blue excitation light (488 nm) was detected and measured by using a FACScalibur flow cytometer (Becton Dickinson,) with a CellQuest software.

### 2.9. Western Blot Analysis

After treating K562 or K562R cells with 6-MITC or STI 571 plus 6-MITC, total proteins were extracted from cells and quantified using a bicinchoninic acid protein assay kit (Bio-Rad Laboratories, Hercules, CA, USA). Proteins were then separated by 10% sodium dodecyl sulfate-polyacrylamide gel electrophoresis (SDS-PAGE) and transferred onto a polyvinylidene difluoride membrane. Blotting was done using various primary antibodies and horseradish peroxidase-conjugated secondary antibodies (1:1000, Santa Cruz Biotechnology, Dallas, TX, USA). The membrane was exposed to enhanced chemiluminescence reagents and analyzed by using a chemiluminescence imaging system (Perkin Elmer, Waltham, MA, USA). Relative protein intensity was estimated by densitometry using the Image J software (Version 1.36b, NIH, Bethesda, MD, USA). The mean values of intensities were normalized to the GAPDH were calculated from at least three independent experiments.

### 2.10. Immunoprecipitation Assay

Cellular proteins were extracted followed by immunoprecipitation overnight at 4 °C with mouse anti-cdc20 monoclonal antibody (Epitomics, Burlingame, CA, USA) and A/G-agarose beads. The precipitated beads were washed with ice-cold cell lysis buffer. The immune complex was subjected to SDS-PAGE gel electrophoresis followed by immunoblotting assay using rabbit anti-BubR1, Mad2 (Bethyl Laboratories, Inc., Montgomery, TX, USA), and cdc23 antibodies (Epitomics).

### 2.11. RNA Interference

Double-stranded, purified RNAi were purchased from Ambion (Austin, TX, USA). Individual sequences targeted for the different gene regions of plk-1 and cdc20 were used to silence gene expression. The sense strand sequences for plk-1 were GAAGAUGCUUCAGACAGAUCCCACU and AGUGGGAUCUGUCUGAAGCAUCUUC. Three cdc20 siRNA were used, and the sequences of sense strand were ACGACAUUUGGCCAGUGGUGGUAAU, AUUACCACCACUGGCCAAAUGUCGU, and CAUGGCCAAGGUGGCUGAACUCAAA (Invitrogen, Waltham, MA, USA). The scrambled RNAi (scRNA) was also used as control (Invitrogen). The RNAi were transfected into cells using lipofectamine^TM^ 2000 (Invitrogen). Cells were plated at 50% confluence and the oligomer-Lipofectamine^TM^ 2000 complex was added. The cells were incubated at 37 °C in a CO_2_ incubator for 24 h. 

### 2.12. Assay for Differentiation Antigens

An indirect immunofluorescence method was used to detect the expression of differentiation-associated antigens on the surface of leukemic cells. Cells collected from day-5 cultures were cultured with primary monoclonal antibodies, washed with PBS, and exposed to FITC-conjugated secondary antibodies, i.e., goat F(ab’)_2_ anti-mouse IgG (Cappel Laboratories, Cochranville, PA, USA). Monoclonal antibodies used included anti-CD14 (BD Biosciences) for monocyte, anti-CD16 (Miltenyi Biotec, Bergisch Gladbach, Germany) for neutrophil, anti-CD235a (BD Biosciences) for erythrocyte, and anti-CD61 (BD Biosciences) for megakaryocyte. FITC conjugated to goat anti-mouse IgG was used to set background thresholds. Flow cytometry with FACScaliber and data analysis using CellQuest ^Pro^ software (Becton Dickinson) were carried out.

### 2.13. Statistical Analysis

The results were expressed as mean ± standard error of the mean (SEM). A comparison in each experiment was performed using an unpaired Student’s T-test or one-way analysis of variance as indicated; a *p*-value < 0.05 indicated statistical significance.

## 3. Results

### 3.1. 6-(methylsulfinyl) hexyl isothiocyanate Inhibited K562 Cell Growth

**Figure 1:**
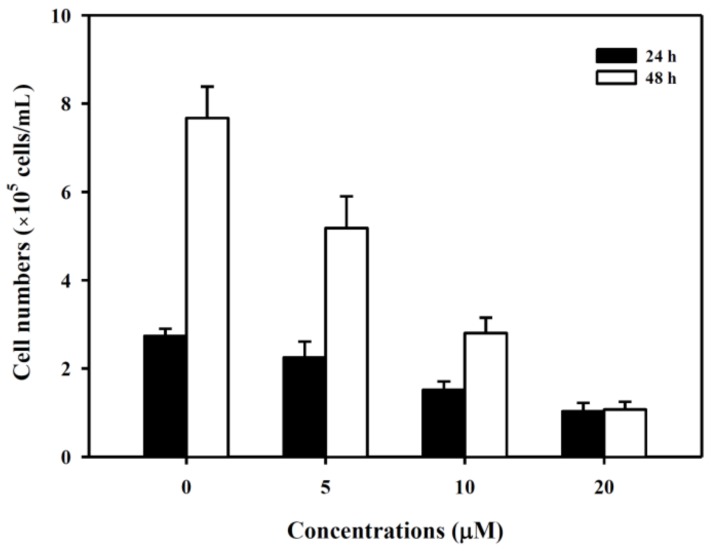
Effect of 6-MITC on the viability of K562 cells. K562 cells were cultured and treated with various concentrations of 6-MITC for 24 h and 48 h. The cell viability was assessed by trypan exclusion assay. Data from three separate experiments are expressed as mean ± standard error of the mean (SEM).

The K562 cells were treated with 6-MITC at 24 h and 48 h with concentrations ranging from 0 to 20 µM; this inhibited the viability of K562 cells in a dose- and time-dependent manner (see Figure 1). The half maximal inhibitory concentration of 6-MITC at 24 h and 48 h were estimated approximately as 13.0 µM and 7.8 µM, respectively.

### 3.2. 6-(methylsulfinyl) hexyl isothiocyanate Did Not Affect the Expression of Bcr-Abl Protein in K562 and K562-Resistant Cells

**Figure 2:**
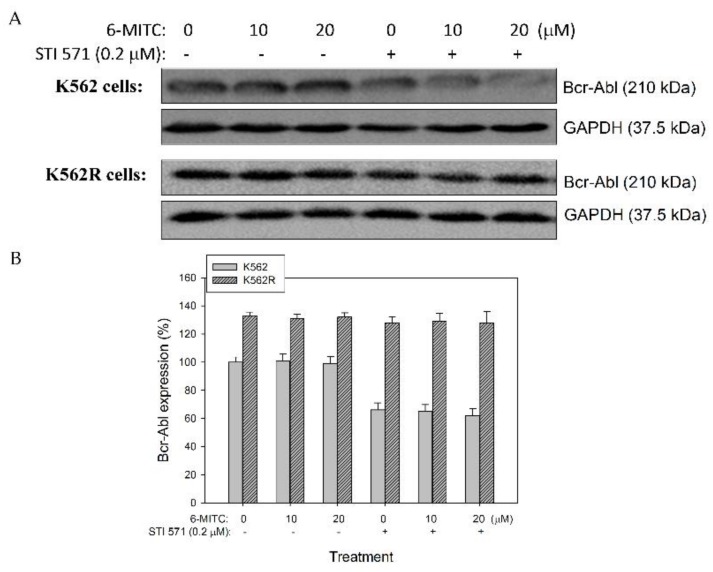
Effect of 6-MITC on Bcr-Abl protein expression in K562 cells and K562R cells. K562 cells and K562R (imatinib/STI 571-resistant) cells were cultured and treated with various concentrations of 6-MITC, STI 571, or STI 571 plus 6-MITC for 48 h. After treatment, the level of Bcr-Abl protein was analyzed by immunoblotting (**A**). The level of Bcr-Abl proteins in the treated cells was expressed relative to that of the control cells (**B**). Data from three separate experiments were expressed as mean ± SEM.

Western blot showed that Bcr-Abl protein expression was altered not only in K562 cells, but also in IM (STI 571)-resistant K562 (K562R) cells after 6-MITC treatment (see Figure 2A). The expression level of Bcr-Abl was reduced in K562 cells after treatment with STI 571 alone or plus 6-MITC, but not in K562R cells (see Figure 2B). The results indicated that 6-MITC inhibited K562 cell growth through mechanisms other than the inhibition of Bcr-Abl expression.

### 3.3. 6-(methylsulfinyl) hexyl isothiocyanate Induced Autophagy in Human K562 Cells

**Figure 3:**
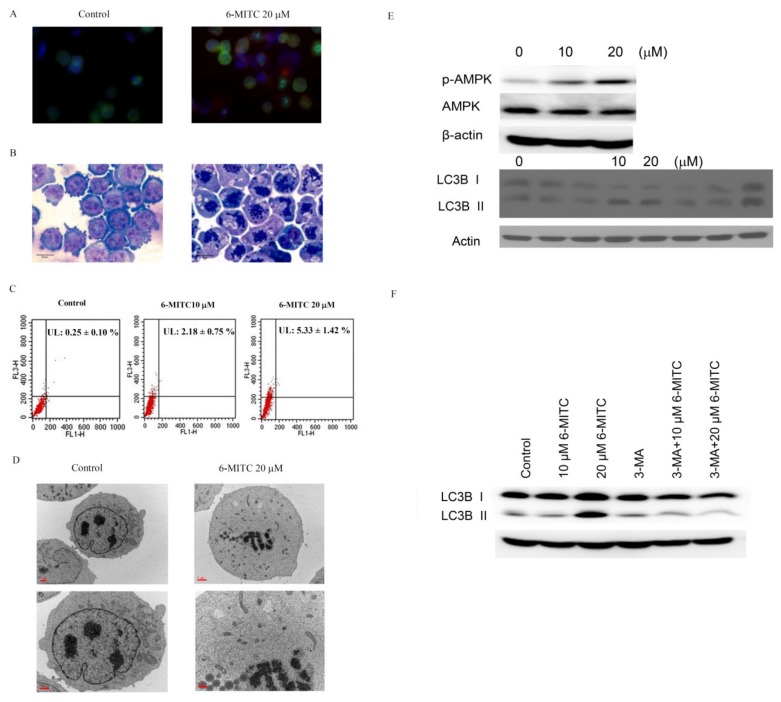
Autophagic features of K562 cells treated with 6-MITC. Cells were treated with or without 20 µM 6-MITC for two days. (**A**) Immunofluorescent stain of α-tubulin (green), γ-tubulin (red), and Hoechst 33258 (blue); (**B**) Liu’s staining for light microscopic observation, photographed at 1000× magnification; (**C**) Acridine orange staining for acidic vesicular organelles using flow cytometry. The expression levels of acridine orange are indicated in the histogram; (**D**) Transmission electron microscopic examination of autophagosome with double-layered membranes, 5000× (upper panels) and 10000× (lower panels) magnification; (**E**) Expression profile of autophagic factors, AMPK and LC3, after 6-MITC exposure. The amounts of AMPK, phosphorylated AMPK, and LC3 were examined using immunoblotting. (**F**) Expression profile of LC3 after treatment with autophagic inhibitor, 3-methyladenine (3-MA), to detect autophagic influx using immunoblotting.

Immunofluorescence staining revealed extensive mitotic arrest with spindle multipolarity after 6-MITC treatment (see Figure 3A). Morphological observation showed extensive small vacuoles accumulation in the cytoplasm of 6-MITC-treated cells (see Figure 3B). We examined the occurrence of autophagy in 6-MITC-treated K562 cells; acridine orange staining using flow cytometry demonstrated that the expression level of acidic vesicular organelles was elevated after 6-MITC administration when compared with the control (see Figure 3C). Using the TEM, we discovered that the accumulated cytoplasmic vacuoles were autophagosomes, double-layered membrane structures containing remnants of organelles (see Figure 3D). The autophagy initiator, adenosine monophasphate (AMP)-activated protein kinase (AMPK), was activated by phosphorylation after 6-MITC administration. The conversion of microtubule-associated protein 1 light chain 3-I (LC3 I) to phosphatidylethanolamine-conjugated LC3 II is one of the characteristic molecular events during autophagy. The conversion of LC3 I to LC3 II was used to validate the development of autophagy (see Figure 3E). The autophagic influx was abolished after treatment with 3-MA, an autophagy inhibitor (see Figure 3F). Taken together, these data suggest that 6-MITC could induce autophagy in K562 cells. The original blots of Figure 3 please see the Figure S1.

### 3.4. 6-(methylsulfinyl) hexyl isothiocyanate Affected Cell Cycle and Arrested in M Phase

**Figure 4:**
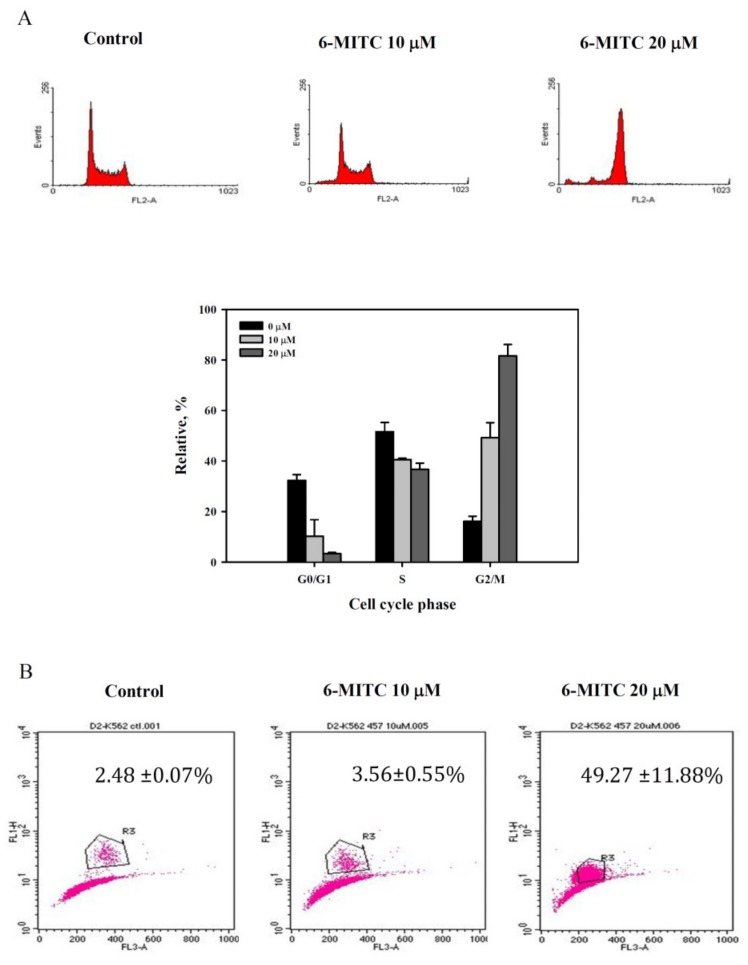
Cell cycle analysis of K562 cells treated with 6-MITC treatment. Cells were treated with 6-MITC at 0-20 μM. (**A**) representative DNA histogram (upper panels) and the expression level of G0/G1, S and G2/M phase (lower panels); (**B**) phosphorylated Histone H3 (pH-3) expression using flow cytometry. The expressed amounts of pH-3 are indicated in the histogram.

**Figure 5:**
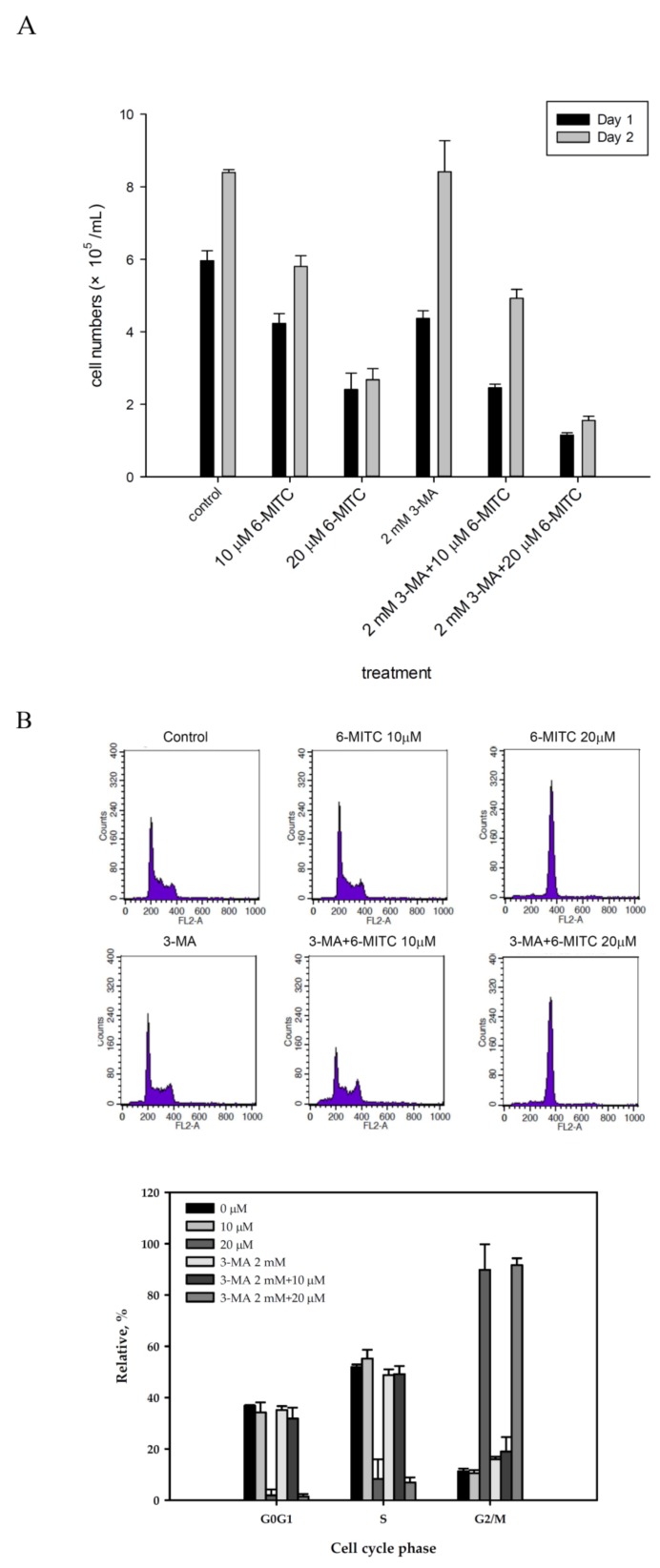
Cell viability and cycle analysis of K562 cells treated with 3-MA, 6-MITC or 3-MA plus 6-MITC. K562 cells were cultured and treated with 6-MITC, 3-MA, or 3-MA plus 6-MITC; cell viability and cell cycle were measured by trypan blue exclusion assay (**A**) and flow cytometry (**B**), respectively. Representative DNA histogram (upper panels) and expression level of G0/G1, S, and G2/M phase are indicated in (**B**).

Cell cycle analysis showed that 6-MITC induced G2/M phase arrest in a concentration-dependent manner (see Figure 4A). Increase in mitotic index and pH-3 expression further verified the specificity of 6-MITC specified at M phase (see Figure 4B). To examine whether 6-MITC induced the autophagy process necessary for cell cycle blockage, 3-MA, as an autophagy inhibitor, was introduced to block the autophagy process, and cell viability, as well as cell cycle, were further analyzed. K562 cell growth was inhibited after 6-MITC treatment, but 3-MA reversed its inhibition effect (see Figure 5A). Cell cycle analysis demonstrated that 3-MA did not influence 6-MITC induced G2/M phase arrest (see Figure 5B). 

### 3.5. Molecular Mechanisms of 6-(methylsulfinyl) hexyl isothiocyanate on K562 Cells

**Figure 6:**
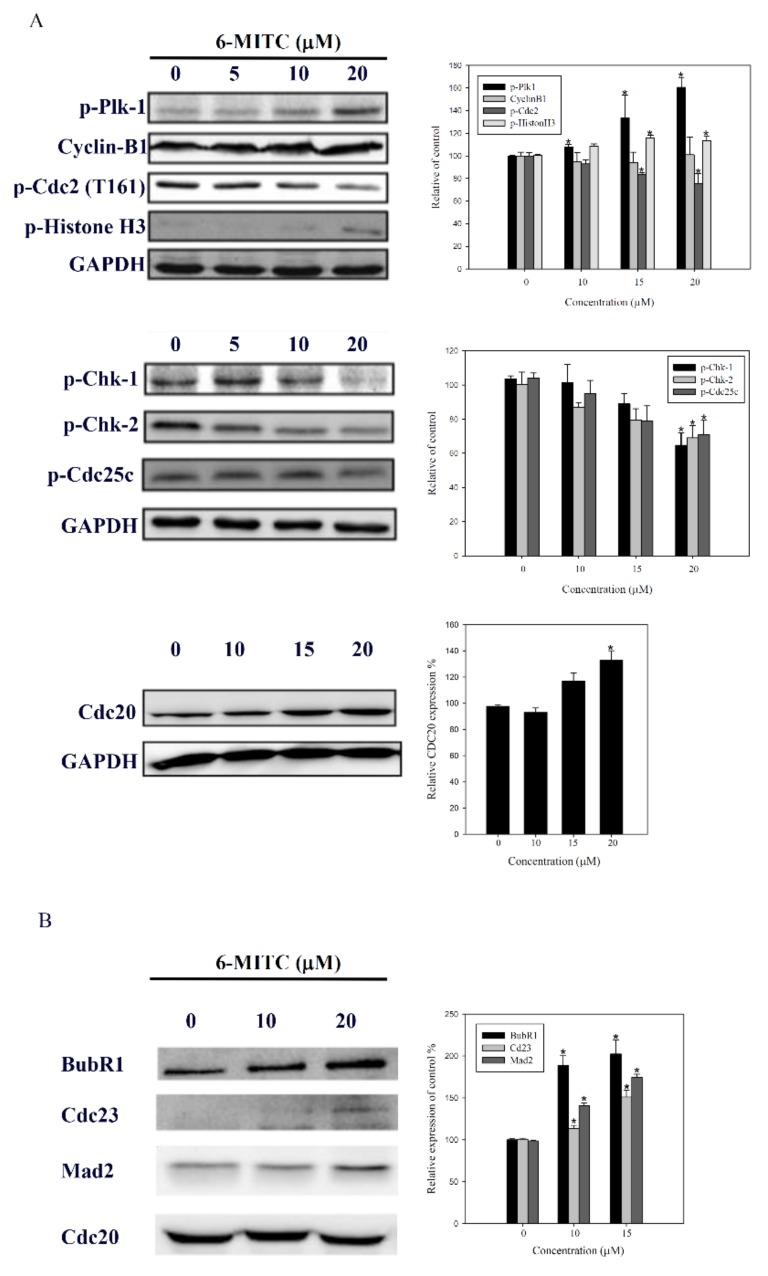
Expression proteins related to mitotic phase in K562 cells. (**A**) K562 cells were treated with 6-MITC for 24 h, the mitosis-related proteins were analyzed by immunoblotting; (**B**) After treatment, cellular lysates were prepared and immunoprecipitated with cdc20. Next, the same amount of proteins were subjected to immunoblot to detect the levels of BubR1, cdc23, and Mad2 proteins. The left parts of each panel are the representative immunoblots of detected proteins. The right parts are the relative level of detected proteins in control cells and cells treated with 6-MITC. Results are mean ± SEM from three independent experiments, and are expressed relative to cells treated with vehicle alone. Significant difference between control cells and cells treated with 6-MITC are indicated by * *p* < 0.05.

**Figure 7:**
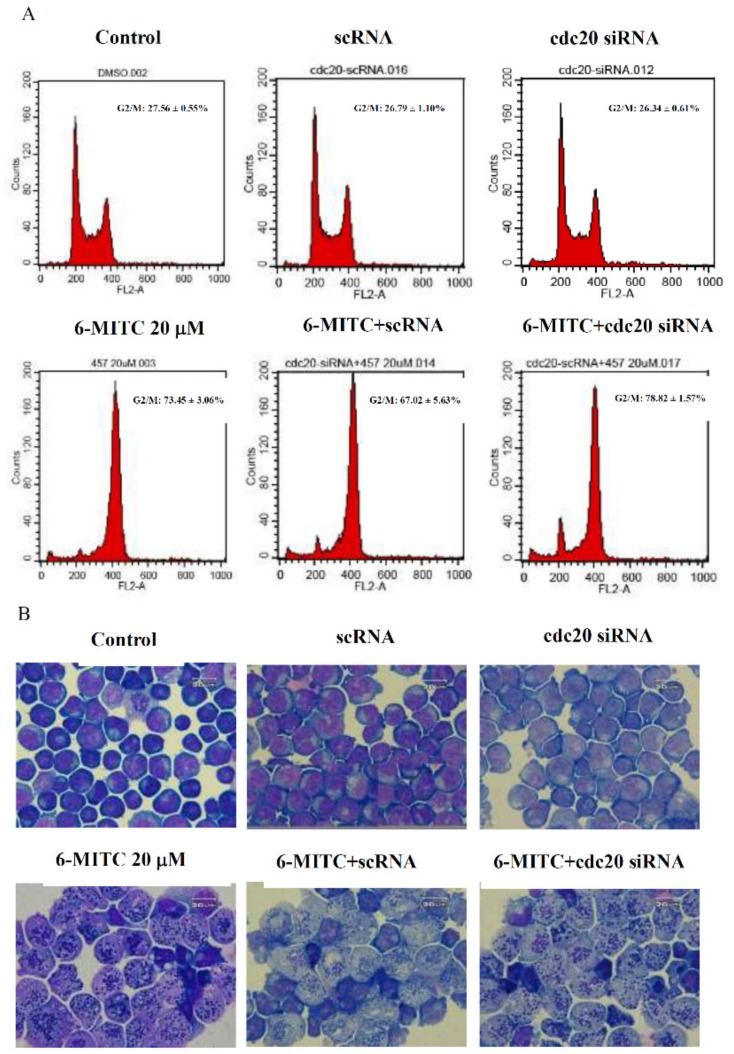
Effect of knockdown of cdc20 on the activity of 6-MITC in K562 cells. (**A**) K562 cells were transfected with cdc20 siRNA or scramble control RNA for 24 h, followed by treatment with vehicle or 6-MITC for 48 h, and cell cycle was determined by flow cytometry. Representative DNA histograms are shown and the percentage of G2/M are indicated in the histogram; (**B**) Morphological examination was performed using Liu’s staining method, and images were photographed at 1000×.

Molecular events related to mitosis suggested the downregulation of p-CHK1, p-CHK2, p-cdc25c, and p-cdc2, and in contrast, the upregulation of cyclin B1, p-cdc20, and p-plk-1 (see Figure 6A). The immunoprecipitation assay further revealed that 6-MITC increased the expression of mitotic checkpoint complex (MCC) molecules, APC8 (cdc23), BubR1, and Mad2, which were bound to the anaphase-promoting/cyclosome (APC/C) complex (see Figure 6B). This evidence indicates that the MCC proteins inhibited the APC/C complex conformational activation and induced mitosis arrest. The knockdown of either cdc20 or scramble interference RNA was unable to block the effect of 6-MITC on mitotic arrest and morphological changes (see Figure 7A,B).

### 3.6. Exclusion of Cell Differentiation by 6-(methylsulfinyl) hexyl isothiocyanate

**Figure 8:**
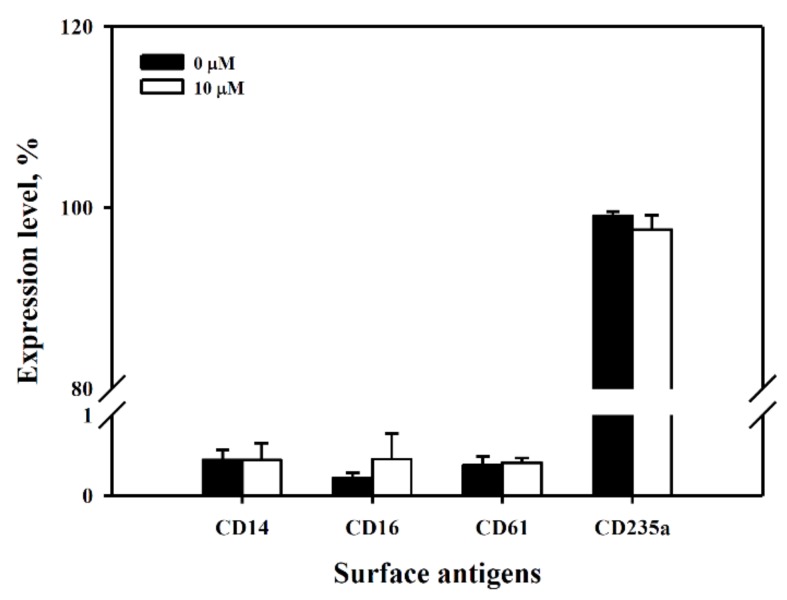
Expression of surface antigens of K562 after exposure to 6-MITC. K562 cells were treated with vehicle or 10 µM 6-MITC for 48 h, the surface antigens CD14 (monocytes), CD16 (granulocytes), CD61 (megakaryocytes), and CD235a (erythrocytes) were detected using flow cytometry. The expression level of these surface antigens on K562 were analyzed by CellQuest Pro software.

To clarify whether 6-MITC could facilitate the differentiation of K562 cells, specific surface antigens CD14, CD16, CD61, and CD235a associated with cell differentiation to monocytes, granulocytes, megakaryocytes, and erythrocytes, respectively, were monitored by flow cytometry. The results showed that 6-MITC did not alter the expression ratios of these surface antigens in treated cells when compare with the control cells (see Figure 8).

## 4. Discussion

In the present study, our evidence suggested that 6-MITC inhibited human leukemia K562 cell growth and induced cell death. The mode of cell death was through two distinct pathways involving autophagy and mitotic arrest. It has been reported that 6-MITC possesses antitumor characteristics through the inhibition of nuclear factor-kappaB (NF-κB) pathway, the disturbance of mitochondrial function, and finally, the induction of cell apoptosis in different cancer cells [27,28]. Previously, we found that 6-MITC acted against tumor cell growth through mitotic arrest, the induction of apoptosis, and the inhibition of the expression of SOX2, a cancer stem cell molecule in human pancreatic cancer cells [26]. Here, we demonstrated a novel mechanism through which 6-MITC suppressed leukemia cell growth by two different modes of cell death. The growth inhibition of leukemia cell by 6-MITC was unrelated to Bcr-Abl protein alteration, because the administration of 6-MITC did not alter the Bcr-Abl protein expression in K562 and K562R cells. Although 6-MITC is isolated from an edible plant, the safety of 6-MITC on non-target cells is also a critical concern. Our previous report showed that 6-MITC had no significant toxicity on human fibroblast cells [23]. The result indicated that 6-MITC is a safe natural compound with cytotoxicity specific to tumor cells.

Autophagy is an evolutionarily-conserved process in cells to maintain homeostasis and cope with nutrient deprivation to meet energy demand. The autophagosomes formed during autophagy fuse with the lysosome to degrade its contents for recycling, and provide nutrients and energy [29]. Several markers or methods have been used to recognize or identify the autophagy process. One of the most powerful methods to detect autophagy is the use of TEM to visualize the autophagic structures. In this study, we used several methods to detect autophagic formation, including morphology to view vacuoles formation, TEM to observe autophagosome formation, and LC3B conversion to detect autophagic influx, as well as upstream phosphorylated AMPK activation. The administration of the autophagic inhibitor, 3-methyladenine (3-MA), reversed the autophagic influx induced by 6-MITC. Previous studies have indicated that a wasabi extract exhibited cytotoxicity on colon cancer cells via apoptosis and autophagy [30]. The mechanisms of autophagy induced by wasabi extract may be through vacuole formation, decreased phosphorylation of AKT and mTOR, and LC3B II formation. Because 6-MITC is the major component of wasabi, we speculate that 6-MITC is the major constituent responsible for the induction of autophagy.

**Figure 9:**
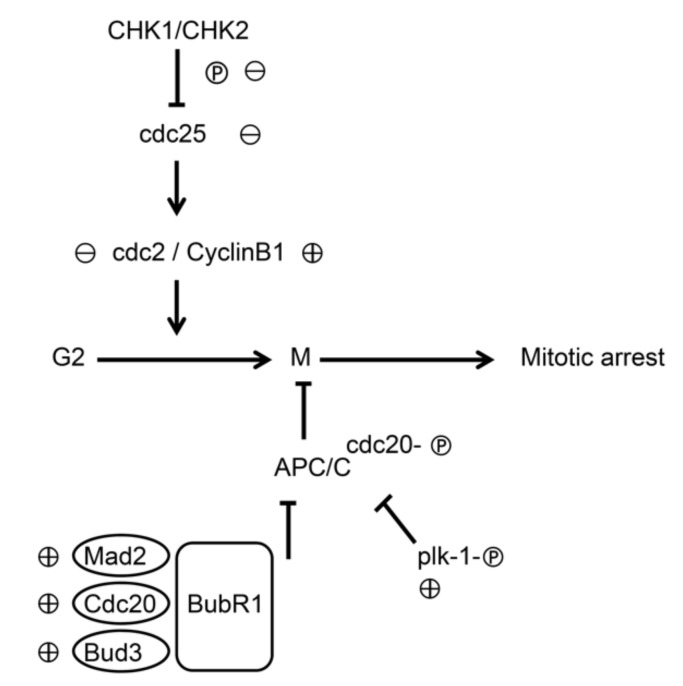
Proposed possible mechanisms of action of 6-MITC in mitotic arrest. Diagram illustration describes the pathways contributing to mitotic arrest by 6-MITC on K562 cells. Circled plus and circled minus indicate the up- and down- regulation of proteins influenced by 6-MITC, respectively.

Mitosis is a dynamic cellular event involving many critical changes such as mitotic spindle formation, chromosome alignment, and segregation, as well as the disintegration of nuclear membrane. The mitotic kinases consisted of CDK1-the homolog of yeast Cdc2, cdc25c, cyclin B, and histone H3. Besides CDK1, other kinases such as Aurora, Polo-like kinase (plk), MCC (Mad2, BubR1, Bub3), and APC/C complex play vital roles in mitotic progression. Our data demonstrated that 6-MITC downregulated upstream mitosis regulators such as p-CHK1 or p-CHK2 and stimulated the expression of cyclin B1, cdc20, APC8 (cdc23), BubR1, Mad2, and p-plk-1. The upregulated MCC bound to APC/C complex to keep APC/C inactive and maintain mitotic arrest without mitotic slippage. The role of mitotic arrest in the development of coexisting autophagy requires further investigation. Taken together, the results presented here provide the possible mechanisms through which 6-MITC provokes mitotic arrest in K562 cells (see Figure 9). It was found that 6-MITC-mediated mitotic arrest involves two G2/M phase-related molecular activity changes. On the one hand, 6-MITC downregulated p-CHK1, p-CHK2, p-cdc25c, and p-cdc2 to inhibit G2/M phase transition. On the other hand, 6-MITC up-regulated the activity of MCC components, and consequently, inhibited the activation of APC/C in the metaphase stage. The actual mechanism through which 6-MITC induced mitotic arrest may involve multiple molecules or signaling pathways.

The inhibition of autophagic activity during mitosis has been reported [31,32]. It was proposed that the inhibition of autophagy serves as a protective mechanism to prevent the unintended loss of organelles and chromosomes in cell and to maintain mitotic homeostasis [33]. In addition, several molecules that regulated G2 to M phase transition abrogate autophagy. CDK1-mediated phosphorylation inhibited autophagy during mitosis [32]. The suppression of Plk-1 activity using BI2536 inhibitor induced autophagy in acute myeloid NB4 and CML K562 cells by downregulating mTOR phosphorylation [34]. Another study revealed the crucial role of Plk-1 in the inhibition of mTOR and promotion of autophagy. However, the role of Plk-1 in modulating autophagy has remained controversial. Targeting the HSP70 protein could inhibit APC/C complex-induced G2/M arrest, along with autophagy inhibition [35]. These results suggest that, under stress, autophagy was inhibited and cell cycle-related molecules were activated to induce mitotic arrest. Nevertheless, chemotherapy drugs that can cause DNA damage could activate ATG5 to modulate autophagy, induce mitotic catastrophe, and cause G2/M arrest [36]. Furthermore, camptothecin or ionizing radiation was able to induce mitotic arrest through MCC activation or G2/M arrest and cell autophagy [37,38]. These findings suggest that autophagy formation enhances stress-induced mitotic phase arrest. The discrepancy in the relationship between autophagy and cell cycle arrest may be caused by different factors. Our results indicate that 6-MITC induces autophagy and mitotic arrest in K562 cells. Pretreatment with an autophagy inhibitor, 3-MA, did not block or enhance 6-MITC-mediated G2/M phase arrest during cell cycle analysis. In other words, the 6-MITC-induced autophagic process is not necessary for cell cycle blockage, but it induces cell death which involves two signaling pathways. The interaction between autophagy and mitotic arrest induced by 6-MITC will be further investigated and elucidated in future studies.

The coexisting mitotic arrest and autophagy by 6-MITC is a unique finding, with potential implications in the development of novel anti-cancer therapeutics, especially for CML. To the best of our knowledge, the wasabi compound 6-MITC might be the first naturally-occurring small molecule to induce concurrent mitotic arrest and autophagy. Further validation by in vivo models and elucidation of its autophagic mechanisms are important.

## 5. Conclusions

In conclusion, 6-MITC inhibited the growth of human CML K562 cells. The compound induced a unique mode of cell death with coexisting mitosis and autophagy, which are regarded as distinct cellular events with counteracting presence. It was found that 6-MITC, a natural compound from *Wasabia japonica*, may have therapeutic potential against CML.

## Supplementary Materials

The following are available online https://www.mdpi.com/2218-273X/9/12/774/s1, Figure S1: The original blots of Figure 3 in the manuscript.
