# Supplementary material for: Wasabi Compound 6-(Methylsulfinyl) Hexyl Isothiocyanate Induces Cell Death with Coexisting Mitotic Arrest and Autophagy in Human Chronic Myelogenous Leukemia K562 Cells
Source: Biomolecules. 2019 Nov 23;9(12):774. doi: 10.3390/biom9120774 (PMC6995613; doi:10.3390/biom9120774)
Supplement: Supplementary file 1 [file biomolecules-09-00774-s001.pdf]

Supplementary files

Wasabi compound 6-(methylsulfinyl) hexyl isothiocyanate induces cell death with co-existing mitotic arrest and autophagy in human chronic myelogenous leukemia K562 cells

Kun-Ming Wu [1,2,3], Hui-Fen Liao [4], Chih-Wen Chi [5,6], Yu Ru Kou [1,*] and Yu-Jen Chen [3,5,7,8,*]

Figure 3: Original blot

p-AMPK and AMPK

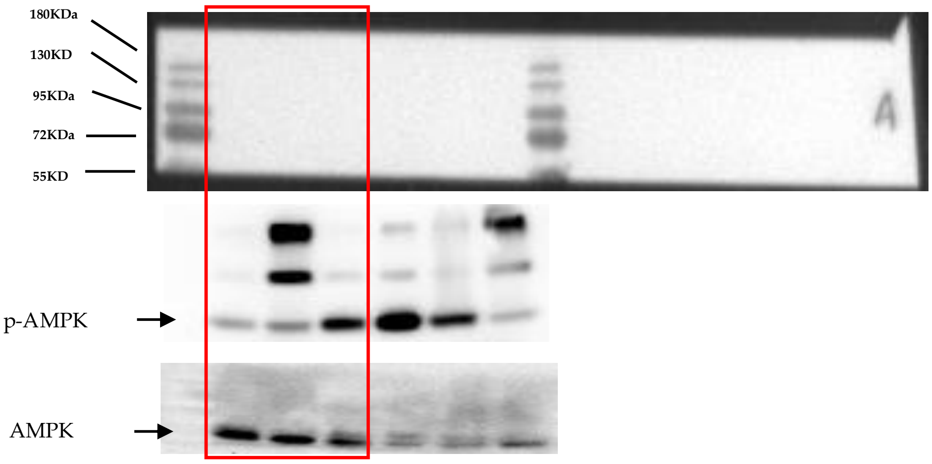

Actin

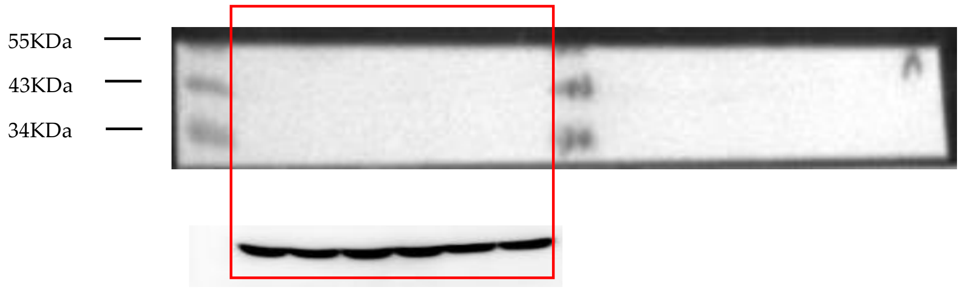

LC3B protein was exposure to x-ray film

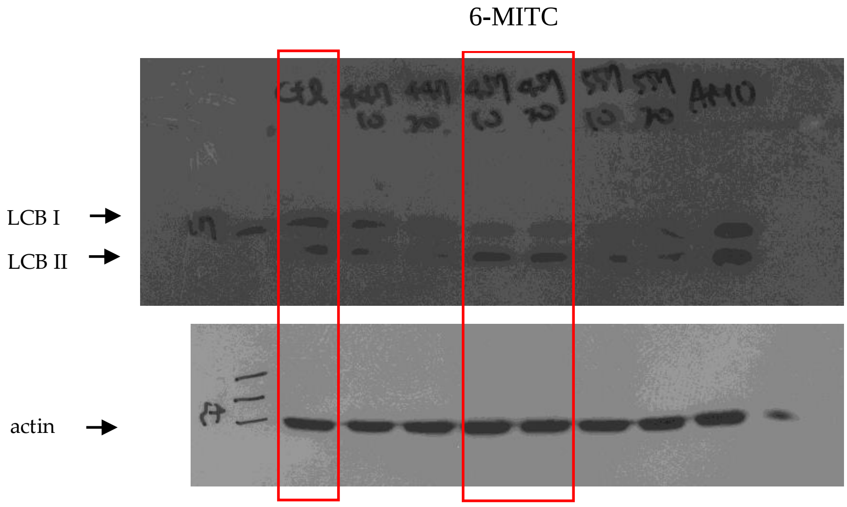

LC3B treatment with autophagic inhibitor, 3-methyladenine (3-MA)

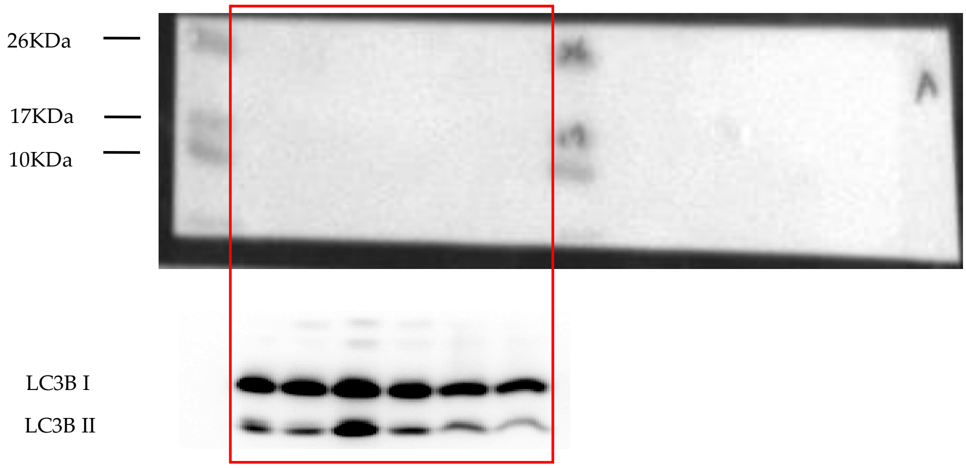

Actin

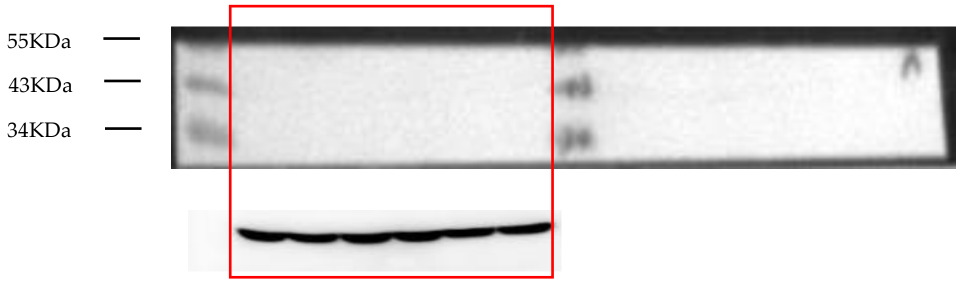

Figure S1. The original blots of figure 3 in the manuscript.
